# Scrotal Skin Metastases Revealing a Prostatic Adenocarcinoma

**DOI:** 10.1155/2020/8826627

**Published:** 2020-09-09

**Authors:** Idriss Ziani, Zayd El Boukili El Makhoukh, Ahmed Ibrahimi, Omar Bellouki, Jihane Benahmed, Yasmine Laraqui, Fouad Zouidia, Hachem El Sayegh, Lounis Benslimane, Yassine Nouini

**Affiliations:** ^1^Department of urological surgery “A”-Ibn Sina University Hospital RABAT-Faculty of Medicine of RABAT, Mohammed V University, Rabat, Morocco; ^2^Department of dermatology and venereology-Ibn Sina University Hospital RABAT-Faculty of Medicine of RABAT, Mohammed V University, Rabat, Morocco; ^3^Department of Anatomopathology and Cytology-Ibn Sina University Hospital RABAT-Faculty of Medicine of RABAT, Mohammed V University, Rabat, Morocco

## Abstract

**Introduction:**

Prostate cancer is the most common cancer in men. Cutaneous metastasis from prostate cancer is an unusual clinical finding. Scrotal skin metastasis revealing a prostate adenocarcinoma is even rarer. *Case Report*. We report the case of a 78-year-old patient, who initially consulted for nonspecific scrotal skin lesions evolving for 4 months. Patient's past history revealed urinary disorders. Physical examination and PSA levels led to perform a prostate biopsy, and the diagnosis of prostate adenocarcinoma was made. Bone scintigraphy showed that the cancer has spread to the bones. Imaging studies showed that the cutaneous lesions were limited to the scrotal wall. Cutaneous metastasis was suspected and was proven on skin biopsy. The patient received second-generation hormone therapy with good clinical and biological outcomes. *Discussion*. Based on literature review of nearly 2,500 skin metastases, we found that only 436 were spreading from the genitourinary tract. Skin metastasis from prostate adenocarcinoma is a rare entity with a low incidence rate (0.36%).

**Conclusion:**

Skin metastases, and especially in the scrotum, are exceptional in prostate cancer. However, in any patient with a prostate adenocarcinoma, nonspecific cutaneous lesions should lead to perform skin biopsy in order to identify and initiate treatment of cutaneous metastases.

## 1. Introduction

According “Cancer Statistics” from 2019 [1], around 174,650 new cases of prostate adenocarcinoma have been reported in men (20%), with very variable mortality rates [2]. In 2018, the highest death rates were recorded in Central America (10.7 per 100,000 inhabitants), followed by Australia and New Zealand (10.2) and Western Europe (10.1) [2]. The 5-year relative survival rate for localized and regional prostate adenocarcinomas is 100%, compared to 30.5% for metastatic ones [3].

The most common metastatic sites are the pelvic lymph nodes, bones, and lungs. Other abdominal and thoracic sites are very rarely reported [4].

Skin metastases are a rare entity with a recorded frequency of 0.7 to 9% of all metastatic malignancies [5].

Despite the fact that prostate cancer is the most common in men, it is exceptionally presented with skin metastases with a published incidence rate of 0.36% [6].

We illustrate through this case report of a rare occurrence of scrotal metastasis revealing prostate adenocarcinoma.

To our knowledge, this is the first observation of scrotal metastases from a prostate adenocarcinoma published in the literature.

## 2. Case Report

A 78-year-old man, with no notable medical history, consults for painless scrotal lesions evolving for 4 months, without any other signs.

**Figure 1:**
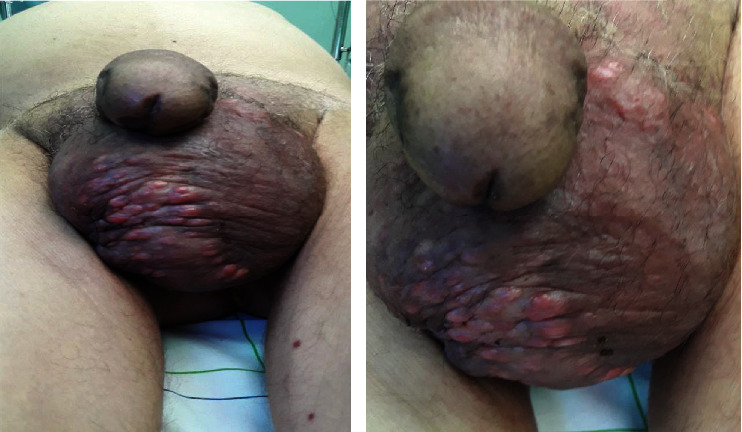
Clinical image of scrotal lesions palpable, firm, soft, and mobile subcutaneous nodules.

The patient reported lower urinary tract symptoms that started 8 months ago, associated to rapidly progressive bones pain, especially in the pelvis, a 10 kg weight loss in 2 months, and deterioration of general condition. Physical examination showed soft, mobile, subcutaneous nodules, and erythematous papules of different sizes (0.5 to 1.5 cm in diameter) in the scrotum (Figure 1).

**Figure 2:**
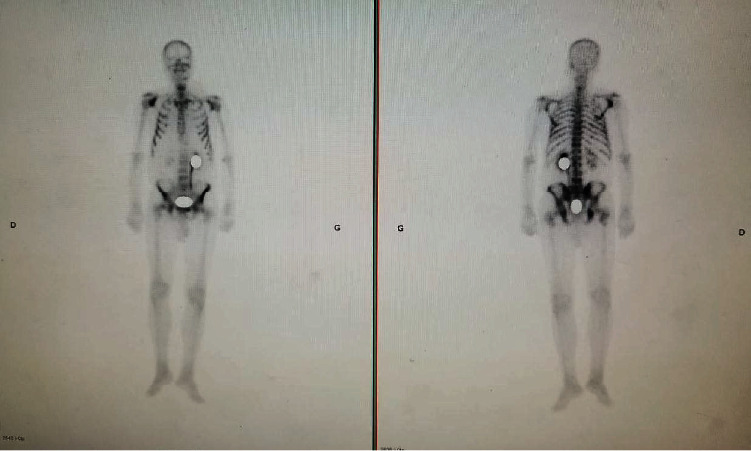
Bone scintigraphy showing several metastatic lesions, including in the lumbar spine.

**Figure 3:**
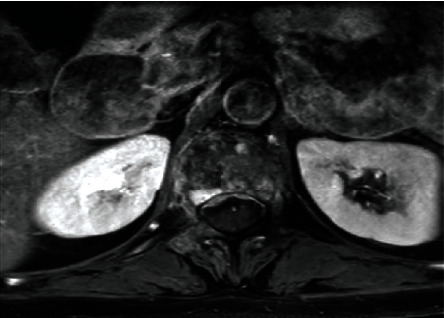
Axial section of an MRI in weighted sequence T1 with gadolinium injection showing irregular contrast uptake of epidural space with heterogeneity of the bone structure vertebral.

**Figure 4:**
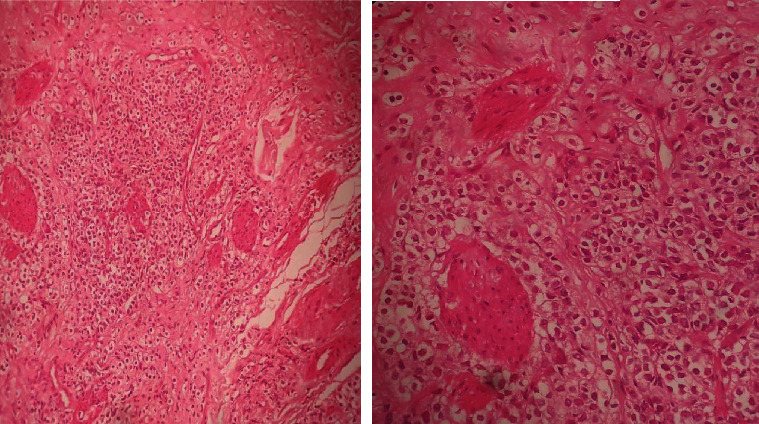
A carcinomatous tumor proliferation located in a small cell stroma, a cytoplasmic shelter, and a monomorphic nucleus sometimes nucleated in the scrotal skin tissue.

**Figure 5:**
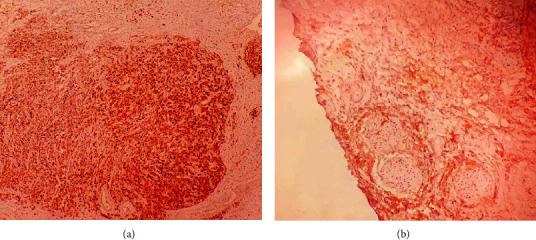
(a) Immunohistochemistry (G ×100): positive labeling with anti-PSA antibody. (b) Immunohistochemistry (×40): positive labeling with anti-PSA antibody with image of perineural neoplastic invasion.

Abnormal digital rectal examination of the prostate with high PSA levels (321 ng/mL) led to perform a prostate biopsy and the diagnosis of prostate adenocarcinoma was made with a Gleason score of 10 (5 + 5) with perineural invasion. Bone scintigraphy revealed bone metastases, which were also identified on the MRI, a lumbar epiduritis was also noted (Figures 2 and 3). Chest and abdominopelvic CT-scan showed no other metastases. At this point of the investigation, scrotal skin metastasis was suspected. The skin biopsy revealed carcinoma cells proliferation organized in a thin stroma. The cells had an abundant cytoplasm with a monomorphic nucleus, some with a nucleolus. This description was compatible with a metastasic scrotal location of a prostate adenocarcinoma. PSA immunohistochemistry (IHC) was positive which confirms the prostatic origin (Figures 4 and 5).

**Figure 6:**
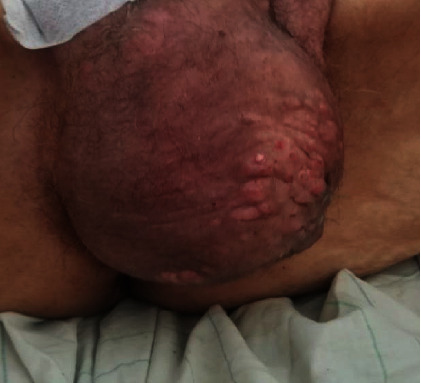
Regression of skin nodules after hormone therapy.

Radiotherapy was urgently performed to treat spinal cord compression. Androgen suppression therapy has been indicated combining an LH-RH analog with second-generation hormone therapy. Abiraterone acetate was used given the high metastatic volume of the cancer. After 3 months, PSA levels dropped to 45 ng/mL and a considerable regression of scrotal skin lesions was noted (Figure 6).

## 3. Discussion

Most common metastatic sites of prostate adenocarcinoma are bones, lungs, liver, and adrenal glands. Skin metastases are rare and usually appear as nodular lesions involving the suprapubic area and the anterior aspect of the thighs [7]. Skin metastases are considered a marker of advanced disease and are correlated with a poor prognosis with an average survival of 6 months [8].

In 2004, Mueller reviewed all the literature on skin metastases of genitourinary origin. Of nearly 2,500 skin metastases, only 436 were from the urogenital tract. He established the incidence rate of skin metastases from prostate cancer at 0.36% [6].

Based on our literature review, we found 59 articles documenting 71 cases of skin metastases. The average age was 67, with extremes of 50 to 81. In 63% of cases, the diagnosis was already known, but it was not specified in 21%. In only 15% of cases, the skin lesions led to the diagnosis of primary prostate cancer [9, 10], which is the case for our patient.

Small cell carcinoma, a very undifferentiated histological form of prostate cancer, is known to have a high metastatic potential with various reported cases of cutaneous metastatic tumors [11, 12].

Four mechanisms of metastasis in prostate adenocarcinoma were identified: local extension from an underlying tumor, implantation in a surgical scar, lymphatic spread, and hematogenous spread.

Skin metastasis is a complex process. Theoretically, the metastatic cells will circulate in the blood or lymphatic vessels and extravasate into dermis in order to proliferate to form the metastatic mass [13].

The clinical presentation can be variable; usually, it appears as erythematous subcutaneous nodules [14, 15]. The unusual, painful erysipelas-like appearance of extensive metastatic lesions was noted [16]. Less often, it may be associated to telangiectasias [17].

Cutaneous metastases are a diagnostic challenge to the pathologists. In prostate adenocarcinoma, skin lesions are reported to be similar to the primary tumor, including dermal invasion with undifferentiated carcinoma cells, often with a clearly visible Grenz area and nuclear atypia characterized by hyperchromasia and prominent eosinophilic nucleoli; connective tissue can also be infiltrated by malignant cells [9, 18]. In addition, immunohistochemistry has been useful to confirm the prostatic origin of atypical metastatic sites. The PSA, a protein normally produced by prostate cells, has been widely used as an immunohistochemical marker, as almost 97% of prostatic metastases can express it [19]. Human Ki-67 protein and CD10 can also be useful as they are often associated with cell proliferation, aggressive migration, and advanced diseases [20, 21].

A particular entity of metastatic skin lesion, described in 1949, is Sister Mary Joseph's nodule. A separate entity defined by nodular metastasis located under the umbilical subcutaneous skin. The nodule is often described as erythematous, firm, elastic, painful, and irregular. It can also appear as an eroded and bloody vascular mass. In 40% of cases, it is a primary malignant tumor. It can also be found in endometriosis (30%) and metastatic skin lesions (20%) [22]. The most common origins of this very rare entity are gastrointestinal and genitourinary tracts.

Therapeutically, many options are reported to manage prostate adenocarcinoma with skin metastases. Mainly, it is a palliative treatment based on tumor excision, radiotherapy, intralesional chemotherapy and treatment of primary neoplasia (i.e., chemotherapy, hormonal, or surgical treatments) [9–22].

In our observation, our patient was treated by second-generation hormone therapy without local treatment of scrotal metastases with good clinical and biological outcomes.

Our case presents the peculiarity of the scrotal localization from a metastatic prostate adenocarcinoma. To the best of our knowledge, it is the first case report in the literature for this particular metastatic site. On the other hand, the fact that skin metastases have appeared before the initial diagnosis of prostate cancer means that dermatologists may be the first to discover the disease. This form of revelation were reported in only 15% of published case reports. Another particular aspect of our case is the regression of the scrotal skin lesions after administration of systemic treatment with second-generation hormone therapy which raises the possibility of systemic preservative treatment for this type of metastasis.

## 4. Conclusion

Skin metastases in prostate cancer are exceptional. The incidence prostate adenocarcinoma with skin metastases is 0.36% of all skin metastases of genitourinary origin. Of those, a scrotal localization is even more exceptional. However, in any patient with a prostate adenocarcinoma, nonspecific cutaneous lesions should lead to perform skin biopsy in order to identify and initiate treatment of cutaneous metastases. Given their rarity in the literature, their management has not yet been codified.

## Data Availability

Citations should appear in the body of the article with a corresponding reference in the reference list.

## References

[1] Siegel R. L., Miller K. D., Jemal A. (2018). Cancer Statistics, 2019. *CA: A Cancer Journal for Clinicians*.

[2] Ferlay J., Ervik M., Lam F. Global cancer observatory: cancer today. Lyon, France: International Agency for Research on Cancer. https://gco.iarc.fr/today.

[3] Cancer Stat Facts (2009–2015). *Prostate cancer*.

[4] Bubendorf L., Schöpfer A., Wagner U. (2000). Metastatic patterns of prostate cancer: an autopsy study of 1,589 patients. *Human Pathology*.

[5] Gül U., Kiliç A., Gönül M., KülcüCakmak S., Erinçkan C. (2007). Spectrum of Cutaneous metastases in 1287 cases of internal Malignancies: a study from Turkey. *Acta Dermato-Venereologica*.

[6] Mueller T. J., Wu H., Greenberg R. E. (2004). Skin metastases of genitourinary malignancies. *Urology*.

[7] Jones C., Rosen T. (1992). Multiple red nodules on lower abdomen. *Archives of Dermatology*.

[8] Wang S. Q., Mecca P. S., Myskowski P. L., Slovin S. F. (2008). Scrotal and penile papules and plaques as the initial manifestation of a cutaneous metastasis of adenocarcinoma of the prostate: case report and review of the literature. *Journal of Cutaneous Pathology*.

[9] Abrol N., Seth A., Chattergee P. (2011). Cutaneous metastasis of prostate carcinoma to neck and upper chest. *Indian Journal of Pathology and Microbiology*.

[10] Thivolet J., Molinie C., Moulin G., Proust J. (1965). Les métastases de la verge au cours du cancer prostatique. *Dermatology*.

[11] Yildirim Y., Akcay Y., Ozyilkan O., Celasun B. (2008). Prostate small cell carcinoma and skin metastases: a rare entity. *Medical Principles and Practice*.

[12] Kaplan M., Atakan I. H., Bilgi S., Inci O. (2007). Case report: subcutaneous metastasis from small cell carcinoma of the prostate. *International Urology and Nephrology*.

[13] Lookingbill D. P., Spangler N., Sexton F. M. (1990). Skin involvement as the presenting sign of internal carcinoma. *Journal of the American Academy of Dermatology*.

[14] Ali N., Ali M., Sabir I., Azhar R., Mansoor S. (2003). Skin metastasis from prostate adenocarcinoma. *Journal of the College of Physicians and Surgeons–Pakistan: Jcpsp*.

[15] Duran E. P., Paradela A., Fariña M. C. (1996). Cutaneous metastases from prostatic carcinoma. *Journal of Surgical Oncology*.

[16] Ng C. S. (2000). Carcinoma erysipeloides from prostate cancer presenting as cellulitis. *Cutis*.

[17] Reddy S., Bang R. H., Contreras M. E. (2007). Telangiectatic cutaneous metastasis from carcinoma of the prostate. *The British Journal of Dermatology*.

[18] Brown G., Kurtzman D., Lian F., Sligh J. (2014). Eruptive nodules of the head and neck: a case report of metastatic prostate cancer. *Dermatology Online Journal*.

[19] Sheridan T., Herawi M., Epstein J. I., Illei P. B. (2007). The role of P501S and PSA in the diagnosis of metastatic adenocarcinoma of the prostate. *The American Journal of Surgical Pathology*.

[20] Dall'Era M. A., True L. D., Siegel A. F., Porter M. P., Sherertz T. M., Liu A. Y. (2007). Differential expression of CD10 in prostate cancer and its clinical implication. *BMC Urology*.

[21] Li R., Heydon K., Hammond M. E. (2004). Ki-67 staining index predicts distant metastasis and survival in locally advanced prostate cancer treated with radiotherapy: an analysis of Patients in radiation therapy Oncology Group Protocol 86-10. *Clinical Cancer Research*.

[22] Gabriele R., Conte M., Egidi F., Borghese M. (2005). Umbilical metastases: current viewpoint. *World Journal of Surgical Oncology*.

